# Biological Activity of Silver(I)-1,10-Phenanthroline Complexes Against *Fonsecaea pedrosoi*: In Silico Predictions, In Vitro Macrophage Interactions and In Vivo Efficacy in *Galleria mellonella*

**DOI:** 10.3390/ph18121819

**Published:** 2025-11-28

**Authors:** Ingrid S. Sousa, Lucas Giovanini, Carolline M. A. Lorentino, Iuri C. Barcellos, Malachy McCann, Michael Devereux, André L. S. Santos, Lucimar F. Kneipp

**Affiliations:** 1 Laboratório de Taxonomia, Bioquímica e Bioprospecção de Fungos (LTBBF), Instituto Oswaldo Cruz, Fundação Oswaldo Cruz, Rio de Janeiro 21040-900, Brazil; ingrid.souza@ioc.fiocruz.br; 2 Laboratório de Estudos Avançados de Microrganismos Emergentes e Resistentes (LEAMER), Instituto de Microbiologia Paulo de Góes (IMPG), Universidade Federal do Rio de Janeiro (UFRJ), Rio de Janeiro 21941-901, Brazil; giovanini@micro.ufrj.br (L.G.); andre@micro.ufrj.br (A.L.S.S.); 3 Department of Chemistry, Maynooth University, W23 F2H6 Maynooth, Ireland; 4 Center for Biomimetic and Therapeutic Research, Focas Research Institute, Technological University Dublin, D08 CKP1 Dublin, Ireland; 5 Rede Micologia RJ, Fundação de Amparo à Pesquisa do Estado do Rio de Janeiro (FAPERJ), Rio de Janeiro 21941-901, Brazil

**Keywords:** metal complexes, antifungal activity, cytotoxicity, cellular interaction

## Abstract

**Background/Objectives:** *Fonsecaea pedrosoi* causes chromoblastomycosis, a neglected chronic subcutaneous mycosis that remains difficult to treat. In this study, we evaluated the toxicity and the antifungal effect of [Ag(1,10-phenanthroline)_2_]ClO_4_ (Ag-phen) and [Ag_2_(3,6,9-trioxaundecanedioate)(1,10-phenanthroline)_4_]·EtOH (Ag-tdda-phen) against *F. pedrosoi* using in silico, in vitro and in vivo approaches. **Methods:** Pharmacokinetic and toxicological parameters were predicted using ADMETlab 2.0. The toxicity of the complexes was assessed using sheep red blood cells, RAW 264.7 macrophage cells, and larvae of *Tenebrio molitor* and *Galleria mellonella*. The effects of these complexes on macrophage adhesion capacity and reactive oxygen species (ROS) production were also investigated using Giemsa staining and dichlorofluorescein diacetate, respectively. In addition, their impact on the survival of *G. mellonella* larvae infected with conidia was evaluated. **Results:** Overall, computational analyses predicted favorable tolerability profiles for both complexes. In vitro assays with red blood cells and macrophages demonstrated that they exhibited selectivity indexes >10 against *F. pedrosoi.* These findings were corroborated by in vivo experiments in which both complexes were injected into insect larvae; the complexes demonstrating good tolerability at concentrations of up to 500 mg/L. Macrophage infection assays revealed that Ag-tdda-phen and Ag-phen markedly reduced the number of intracellular conidia. These effects appear to be associated with oxidative stress, as macrophage production of ROS significantly increased following treatment with the complexes. Furthermore, Ag-tdda-phen improved the survival of *G. mellonella* larvae infected with *F. pedrosoi*, demonstrating a protective effect. **Conclusions:** Collectively, our findings support the notion that silver(I)-phen derivatives represent promising candidates for the development of therapeutic options against CBM infections caused by *F. pedrosoi*.

## 1. Introduction

*Fonsecaea pedrosoi* is a melanized fungus and the main causative agent of chromoblastomycosis (CBM), a disease that affects cutaneous and subcutaneous tissues [1,2]. CBM is considered by the World Health Organization (WHO) as a neglected tropical disease [3]; it occurs all over the world but is prevalent in tropical and subtropical regions [4,5]. This disease causes distinct chronic and polymorphic lesions that are verrucous, nodular, plaque-like, squamous, and ulcerative [1,6]. The severe form of CBM is associated with secondary bacterial infections, elephantiasis-like lymphedema and neoplastic transformation of lesions that may provoke incapacity of the affected limb [6,7]. There is no gold standard treatment for CBM, and most current therapies have limited efficacy, numerous side effects, and high costs, which hinder access in endemic areas [2,8]. Therefore, CBM represents a public health concern and highlights the urgent need for alternative antifungal agents.

Several studies have shown that 1,10-phenantholine (phen)/1,10-phenantholine-5,6-dione (phendione) ligands coordinated to different transition metals can act on tumor and microbial cells including fungi [9,10,11,12,13]. The mechanisms of action of these complexes on fungal cells have not yet been fully elucidated, but they appear to be involved in several crucial events, including disruption of the cell membrane and internal organelles, impairment of mitochondrial function, and nuclear fragmentation [9,14]. Indeed, studies indicate that metal-based compounds can exert multimodal actions, positioning them as viable alternatives to conventional antifungal drugs and as promising candidates for combination therapies [9,15]. In addition, studies carried out by our group revealed that, in general, in in vitro cell lines, as well as in vivo models using *Galleria mellonella* larvae and mice, phen/phendione and its metal-based complexes were well tolerated [16,17].

This set of complexes has also been the focus of our research in recent years, particularly regarding CBM-associated fungi [18,19,20]. Our previous studies with *Phialophora verrucosa*, another CBM-associated fungus, revealed that [Ag(phendione)_2_]ClO_4_ and [Cu(phendione)_3_](ClO_4_)_2_.4H_2_O were able to inhibit its growth, filamentation, ergosterol content, and metallopeptidase activity and protect *G. mellonella* larvae from fungal infection [18,19]. In addition, we demonstrated that *F. pedrosoi* was sensitive to 13 different metal-based compounds, including copper(II)-, manganese(II)- and silver(I)-complexes coordinated with either phen or phendione [20]. In that study, the most effective complexes against *F. pedrosoi* were [Ag(1,10-phenanthroline)_2_]ClO_4_ (Ag-phen) and [Ag_2_(3,6,9-trioxaundecanedioate)(1,10-phenanthroline)_4_]·EtOH (Ag-tdda-phen), which exhibited minimum inhibitory concentration (MIC) and minimum fungicidal concentration (MFC) values of 1.2/5.0 µM and 0.6/2.5 µM, respectively [20]. Additionally, our previous studies revealed that both complexes were able to inhibit the growth of *F. pedrosoi* even under biofilm conditions, showing greater antimicrobial activity than itraconazole, the antifungal used clinically for CBM treatment [20]. The association of non-inhibitory concentrations of Ag-tdda-phen with itraconazole drastically reduced the viability of the *F. pedrosoi* biofilm as well as the extracellular matrix amount, which was also affected by the Ag-phen [20]. Both silver(I) complexes were able to inhibit metallo- and aspartic peptidase activities, the transition from conidia into mycelium, as well as fungal melanin production. The silver(I) complexes also induced *F. pedrosoi* production of reactive oxygen species (ROS), indicating that oxidative stress may be involved with their antifungal activity [20].

In this work, we evaluated the pharmacokinetic and toxicological profiles of Ag-phen and Ag-tdda-phen using the ADMETlab 2.0 software, as well as their toxicity in vitro in macrophage cells and in vivo in both *Tenebrio molitor* and *G. mellonella* larvae. Furthermore, following infection with *F. pedrosoi* conidia, we investigated the effects of these complexes on macrophage adhesion capacity and ROS production, as well as on the survival of *G. mellonella* larvae.

## 2. Results and Discussion

### 2.1. Prediction of Compounds Pharmacokinetic and Toxicological Parameters

**Figure 1:**
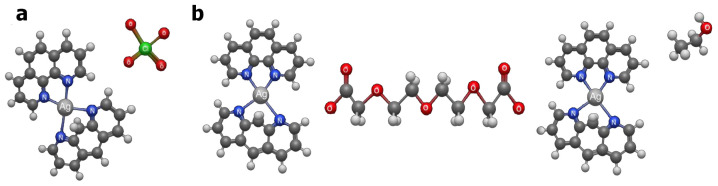
Three-dimensional structural models of the Ag–phen (**a**) and Ag–tdda–phen (**b**) complexes generated using Avogadro (version 2.1.100.0).

Pharmacokinetic and toxicological parameters for the test complexes Ag-phen and Ag-tdda-phen (structural formulas shown in Figure 1) were predicted and assessed, with itraconazole included as a reference therapeutic agent. Regarding drug-likeness parameters, Ag-phen showed no violation of Lipinski’s rules, while for Ag-tdda-phen and itraconazole, more than two of these properties were out of range (Table 1). Such violations include having more than five hydrogen bond donors, more than ten hydrogen bond acceptors, molecular weights >500 Daltons, or a partition coefficient (logP) greater than 5 [21]. When Pfizer’s rules were considered, both complexes and itraconazole obeyed all established criteria (logP > 3 and topological polar surface area (TPSA) <75) (Table 1) and therefore had acceptable predicted toxicological risks [22].

Absorption parameters indicated that both complexes exhibit low human intestinal absorption (HIA) and low fractions of the dose reach the systemic circulation (F20% and F30%), suggesting reduced oral bioavailability (Table 1). In contrast to the results obtained for itraconazole and those predicted by the SwissADME software (http://www.swissadme.ch/, accessed on 1 April 2025), the phen derivative [Ag(phendione)_2_]ClO_4_, was predicted to have high oral absorption [17]. Although oral absorption is an essential prerequisite in in silico ADMET studies, some promising drugs may exhibit low HIA when alternative routes of administration are feasible, including subcutaneous injection or topical drug delivery [23,24]. Topical drugs can also be effective especially in combination with antifungal agents and be used as adjuvant therapies for CBM [1]. Tendolkar et al. [25] demonstrated that local application of copper sulfate combined with oral itraconazole strongly reduced phaeohyphomycotic ulcer caused by *P. verrucosa.*

Other models for predicting absorption properties were studied, but with conflicting results. Both silver(I) complexes have low permeability for colon adenocarcinoma (CaCo-2) cells, but they showed excellent permeability when Maden Darby Canine Kidney (MDCK) cells were used as experimental models (Table 1). Interestingly, considering the distribution parameters, the compounds presented plasma protein binding (PPB) values <90%, which are better than itraconazole; the low capacity to bind to plasma proteins can impact on the bioavailability of the drug and consequently increase its therapeutic index. This parameter was corroborated since both compounds presented high volume of distribution (VD) values (optimal range 0.04–20 L/kg), which is indicative of adequate tissue distribution. Our data revealed that Ag-tdda-phen was unable to penetrate the blood–brain barrier (BBB), while compound Ag-phen and itraconazole had moderate and high abilities to reach the central nervous system, respectively (Table 1). It is important to consider that CBM is essentially a subcutaneous mycosis, and the lack of BBB penetration prevents side effects on neural tissues. Both compounds presented favorable results regarding the fraction unbound (Fu) parameter since they were unable to bind to serum proteins in plasma (values < 5), facilitating their membrane crossing and better diffusion.

Regarding metabolism, in contrast to itraconazole, which inhibited cytochrome P450 isoenzymes (CYP3A4, CYP2C9, and CYP3A4), both compounds demonstrated favorable profiles without acting as CYP inhibitors (Table 1). Ag-phen and Ag-tdda-phen showed the same behavior as that described for [Ag(phendione)_2_]ClO_4_ when the SwissADME software was used, which revealed their inability to inhibit cytochrome P450 enzymes, which is indicative of a lower risk of metabolic drug interactions [17]. In addition, excretion data indicated that both compounds exhibited low clearance (CL) values (<5 mL/min/kg) while itraconazole had a moderate CL (Table 1). Although slow elimination contributes to maintaining drug concentrations and therapeutic effects, it can lead to drug accumulation in the body and may occasionally be toxic [26]. Both compounds as well as the antifungal agent presented low half-life (T½) values, suggesting the probability of a short drug half-life.

When toxicity parameters were evaluated, we observed that, unlike itraconazole, Ag-phen and Ag-tdda-phen were unable to block human ether-à-go-go-related gene (hERG), suggesting that they cannot cause cardiac side effects such as arrhythmia and even sudden death. However, human hepatotoxicity (H-HT) and drug-induced liver injury (DILI) predictions indicated that both the test complexes and itraconazole have the potential to cause liver injury (Table 1). Ag-tdda-phen showed moderate toxicity in the AMES test, but Ag-phen and itraconazole showed high mutagenic potential. In addition, Ag-tdda-phen presented low rat oral acute (ROA) toxicity, one of the most important parameters for assessing the safety of drug candidates [27]. Conversely, Ag-phen and itraconazole showed high and moderate ROA toxicity, respectively. The two silver(I) complexes and itraconazole exhibited the same behavior regarding skin sensitization (SS) and carcinogenic potency (CP) parameters, indicating that they can cause allergic contact dermatitis and be classed as being carcinogenic (Table 1).

Taken together, these data revealed that, although only Ag-phen was approved under the Lipinski rules, both complexes were approved under the Pfizer rules. Even though the silver(I) complexes had limitations regarding oral absorption, they exhibited favorable metabolic and distribution profiles. It is important to highlight that Ag-tdda-phen performed best in terms of parameters such as BBB, the AMES test, and ROA toxicity. Overall, based on the predictions from ADMETlab 2.0, Ag-tdda-phen presented a more favorable toxicological profile, offering potential as a topical antifungal agent.

### 2.2. Toxicity of Test Complexes: In Vitro and In Vivo Approaches

**Figure 2:**
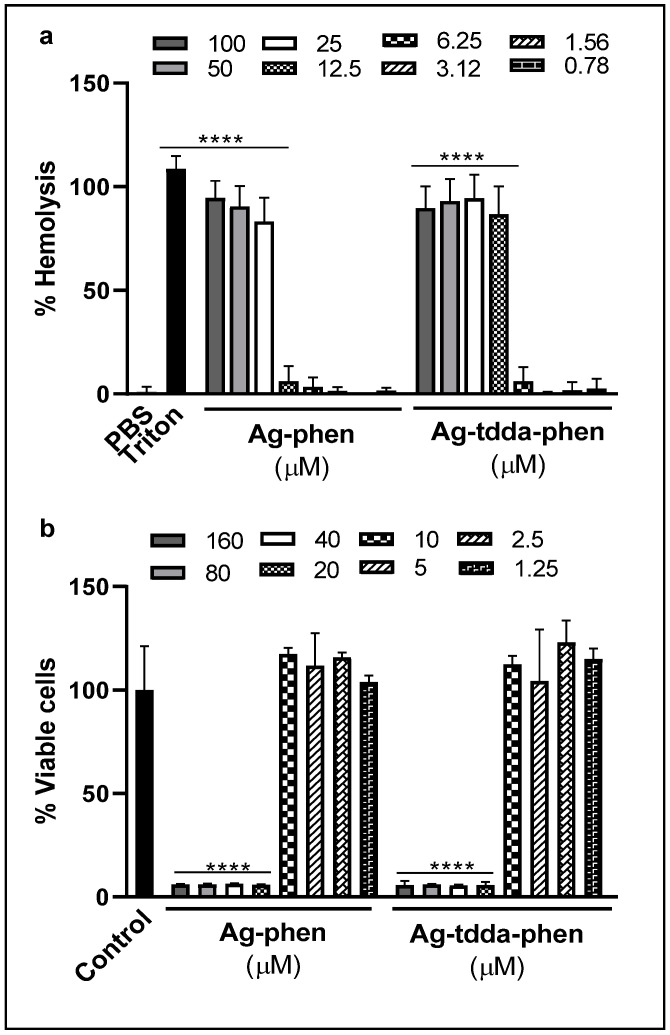
Cytotoxicity of compounds in vitro. (**a**) Red blood cells were incubated in the absence (control: phosphate-buffered saline (PBS), pH 7.2) or in the presence of different concentrations of Ag-phen and Ag-tdda-phen for 24 h and the hemolytic activity determined after reading at 415 nm. Triton X-100 was added to regarded % hemolysis. (**b**) RAW macrophage cells were treated with different concentrations of both compounds for 20 h and cellular viability was determined using the MTT assay, as detailed in the Material and Methods. Untreated macrophage cells were used as a control. Absorbance values were converted to percentages relative to the control. **** *p* < 0.0001.

The cytotoxicity test was performed using the hemolytic assay. The results showed that red blood cells remained viable (>90%) at concentrations up to 10-fold higher than the MIC values of both metal complexes, i.e., ≤12.5 µM and ≤6.25 µM for Ag-phen and Ag-tdda-phen, respectively (Figure 2a). The results showed that, after 24 h of incubation, the hemolytic activity of both complexes was higher than that of other phen/phendione derivatives, including [Ag(phendione)_2_]ClO_4_, which did not show significant hemolysis up to 62.5 µM [17]. However, the hemolytic rate was lower than that of amphotericin B, a commercial antifungal agent, which was able to lyse ~60% of red blood cells at concentration of 8 µg/mL after only 1 h of incubation [28]. Based on these findings, the concentration capable of reducing fungal viability by 50% (CC_50_) was established for both complexes and the selectivity index (SI), a parameter to select whether a compound is safe considering its toxicity to host cells and its antimicrobial activity, was calculated [29]. The complexes Ag-phen and Ag-tdda-phen presented SIs >10 for *F. pedrosoi* with values of 27.4 and 32.3, respectively (Table 2). Our results were similar to those described for [Ag(phendione)_2_]ClO_4_, which presented SI values >40 for *Cryptococcus gattii* and *C. neoformans* [17]. Furthermore, a related Cu(II) phen-based complex, Cu(theo)_2_phen(H_2_O)·5H_2_O (theo = theophylline) showed SI values ranging from >16 to >64 for *Candida* species when blood cells were also used as an in vitro experimental model [30].

In addition, we evaluated the cytotoxicity of the two silver(I) complexes using macrophage cells, which allowed us to determine the absence of toxicity after treatment with concentrations equal to or less than 10 μM of both compounds (Figure 2b). Using CC_50_ values >10 μM for both complexes, SI values of >16.1 and >32.2 were established for Ag-phen and Ag-tdda-phen, respectively (Table 2). Consequently, the CC_50_ values for the complexes were >10 μM, and SI values of >16.1 and >32.2 were determined for Ag-phen and Ag-tdda-phen, respectively (Table 2). These values are notably higher than those observed for the *Candida haemulonii* complex, which showed mean SI values ranging from >1.0 to <3.0 in cytotoxicity assays using the A549 lung epithelial cell line and also the 3-[4,5-dimethyl-thiazol-2-yl]-2,5-diphenyltetrazolium bromide (MTT) assay [14]. Corroborating our findings, Mello et al. [31] demonstrated that *Scedosporium minutisporum*, even when tested with A549 cells, exhibited a favorable SI (>10) for Ag-tdda-phen.

Overall, our data revealed that SI values >10 for Ag-phen and Ag-tdda-phen, using red blood and macrophage cells, confirms that both silver(I) complexes have higher selectivity for *F. pedrosoi* than for host cells, indicating their potential as promising drugs candidates.

**Figure 3:**
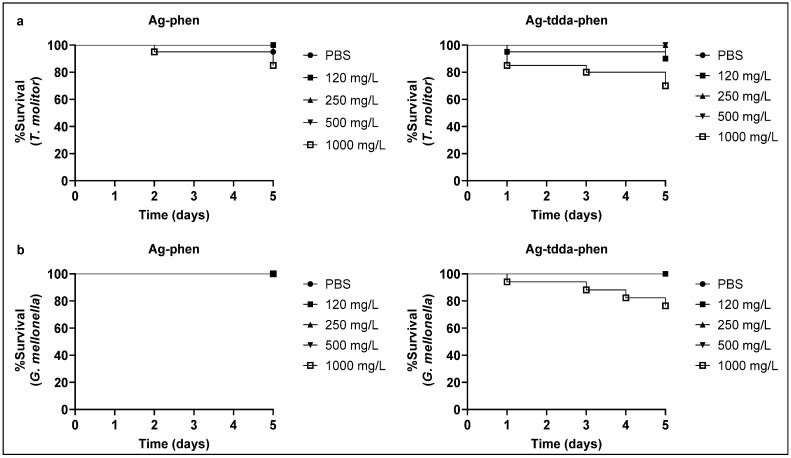
Toxicity of compounds in vivo. Groups of 10 (**a**) *T. molitor* and (**b**) *G. mellonella* larvae were treated with the compounds at 120–1000 mg/L (1000 mg/L corresponds to 1785.7 µM for Ag-phen and 892.8 µM for Ag-tdda-phen) and incubated for 5 days at 37 °C. Larvae inoculated with PBS served as controls. Mortality was assessed every 24 h and was defined as the presence of dark pigmentation and the absence of movement in response to mechanical stimulation.

The in vivo toxicity assay was performed using *T. molitor* and *G. mellonella* larvae since these insect models offer several advantages, including the absence of ethical restrictions, ease of maintenance, short life cycle, and an innate immune system that resembles that of vertebrates [32,33]. At 5 days, our data showed that *G. mellonella* larvae were 100% viable even after injection of 1000 mg/L (1785.7 µM per larva) Ag-phen, while 85% of *T. molitor* larvae remained viable (Figure 3). Under the same conditions, after treatment with Ag-tdda-phen (1000 mg/L [892.8 µM] per larva), both invertebrate larvae showed ~70% viability. It is important to note that the viabilities of *T. molitor* and *G. mellonella* larvae were not affected by either complex at concentrations of up to 500 mg/L, corresponding to approximately 30 mg/kg and 20 mg/kg of larvae for Ag-phen and Ag-tdda-phen, respectively. These findings suggest that the complexes were well tolerated by both invertebrate models as previously shown for other metal-based phen compounds [16,30]. The toxicity of both complexes was previously studied using *G. mellonella* larvae with survival monitoring up to 72 h [16]. As shown by the present results, additional toxicity testing using the *T. molitor* invertebrate model confirmed the high tolerance to both silver(I) complexes, which was in agreement with the in silico assay findings. In addition, the determination of non-inhibitory concentrations was also crucial for conducting the subsequent experiment evaluating the effect of the complexes on in vivo infection, for which *G. mellonella* was selected as the model organism.

### 2.3. Effect of Compounds on Macrophages Infected with F. pedrosoi

**Figure 4:**
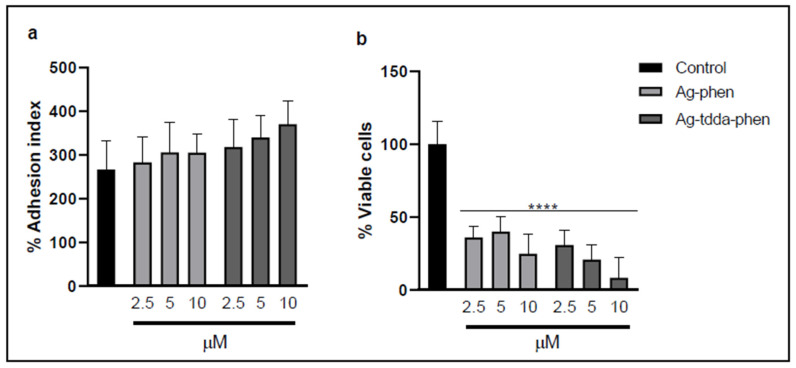
Effects of Ag-phen and Ag-tdda-phen on the interaction between *F. pedrosoi* and macrophages. The cell suspension (1 × 10^5^) was incubated for 20 h in the absence (control) or in the presence of different concentrations of Ag-phen and Ag-tdda-phen. (**a**) The adhesion index was determined after infection of macrophages with *F. pedrosoi* conidia at a 1:10 ratio for 1 h, followed by treatment for an additional 20 h with non-cytotoxic concentrations of both compounds. (**b**) The number of viable conidia was quantified after macrophage lysis, followed by plating on Sabouraud Dextrose Agar (SDA) medium and incubation for 6 days at 26 °C. The inhibition ratio was determined using a colony-forming unit assay and expressed as a percentage relative to the control values. **** *p* < 0.0001.

Considering that fungal–host cell interaction is also an important factor in the pathogenesis of *F. pedrosoi*, experiments to evaluate the effect of the silver(I)-phen complexes on conidia after in vitro interaction with RAW 264.7 murine macrophages were performed. Under these conditions, non-cytotoxic concentrations of both complexes (≤10 µM) did not inhibit the adhesion of *F. pedrosoi* conidia to macrophages (Figure 4a). However, when macrophage killing activity was assessed, conidia pretreated with either complex exhibited markedly increased susceptibility to macrophage-mediated killing (Figure 4b). At a concentration of 10 µM, Ag-phen and Ag-tdda-phen inhibited approximately 75% and 90% of the endocytosed conidia, respectively. Our group demonstrated a similar effect using silver(I) and copper(II) phendione complexes, [Ag(phendione)_2_]ClO_4_ and [Cu(phendione)_3_](ClO_4_)_2_.4H_2_O, whereby each complex did not affect cellular adhesion but significantly reduced the viability of *P. verrucosa* following interaction with THP-1 macrophages derived from human monocytes [19]. It is worth noting that fungal cells previously exposed to compounds with antifungal activity may still adhere to macrophages even after losing viability. This apparent discrepancy can be explained by the fact that adhesion depends primarily on the structural integrity of the fungal cell wall rather than on metabolic activity. The cell wall components, including mannans, β-glucans, and surface adhesins, remain intact for some time after cell death and can still interact with macrophage receptors such as Dectin-1 and Toll-like receptors [34,35]. Therefore, although the treated fungal cells were no longer able to form colonies in colony-forming unit (CFU) assays, their preserved cell wall structures likely supported adherence to host cells. Guirao-Abad et al. [36] showed that although *Candida albicans* treatment with micafungin has an antifungal effect as expected, it potentiates the immune response, improving the interaction of human macrophages, probably through the unmasking of β-glucans on the cell wall surface. These findings suggest that the compounds may affect fungal viability without immediately disrupting the cell surface, allowing for recognition and attachment by macrophages.

**Figure 5:**
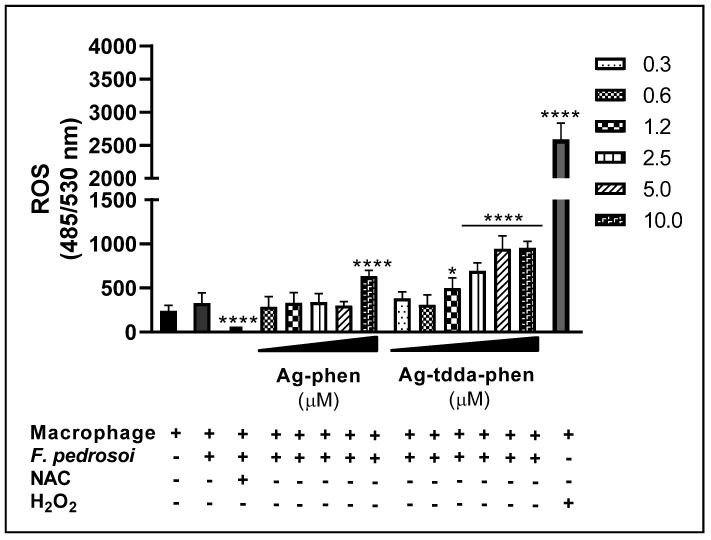
Effects of Ag-phen and Ag-tdda-phen compounds on the reactive oxygen species (ROS) production of macrophages infected with *F. pedrosoi*. Uninfected and untreated *F. pedrosoi*-infected (control system) macrophages and those treated with non-inhibitory concentrations of the compounds were incubated with the probe dichlorofluorescein diacetate (10 µM) and monitored for 2 h. Hydrogen peroxide (H_2_O_2_, 1 mM) was used as a positive control for ROS production, and the antioxidant *N*-acetylcysteine (NAC, 15 mM) was added as a ROS inhibitor. **** *p* < 0.0001 and * *p* < 0.05 denote significant differences in comparison with the control systems.

Since these complexes were added to the macrophage–fungus interaction medium, the observed reduction in fungal growth may also result from their action on the host cell, potentially enhancing the microbicidal capacity of macrophages. In this context, ROS production was evaluated to determine whether conidial susceptibility is also linked to the direct cytotoxic activity of macrophage, given that the respiratory burst represents one of the key mechanisms underlying the antimicrobial immunity of phagocytic cells [37,38]. Our experiments revealed that the macrophage ROS level was not significantly affected after 2 h of fungal cell incubation (Figure 5). On the other hand, the antioxidant *N*-acetylcysteine (NAC) drastically reduced the signal (~44-fold) compared to the control system, indicating that ROS may be involved in this process (Figure 5). In fact, NAC is known to be a ROS scavenger and has been commonly used for studying the role of ROS in several biological and pathological events [37]. The capability of macrophages to produce ROS was also corroborated when the present system was treated with hydrogen peroxide (positive control), which increased fluorescence by approximately 8-fold. Our data revealed that Ag-phen, at the highest concentration tested (10 µM), increased ROS production by approximately 2-fold compared to the control system. With Ag-tdda-phen, ROS production increased by 1.5- and 2-fold at concentrations equal to 1.2 and 2.5 µM, and approximately 3-fold at concentrations of 5 and 10 µM (Figure 5).

These macrophage ROS results suggest a correlation with the data from the killing experiment that showed a drastic inhibition in the number of conidia following treatment with the silver(I) complexes (Figure 4b). Thus, the action of both complexes could be attributed, in part, to their antifungal activity, which reduces the number of intracellular conidia and/or modulates macrophage mechanisms related to antimicrobial immune responses. Interestingly, these inhibitory and immunomodulatory effects were also observed following treatment of RAW 264.7 macrophages infected with *Toxoplasma gondii* with biogenic silver nanoparticles. Similarly, the authors of [39] showed that silver nanoparticles were able to reduce parasite proliferation in macrophage cells and induce ROS production.

### 2.4. Effect of Compounds on G. mellonella Larvae Infected with F. pedrosoi

**Figure 6:**
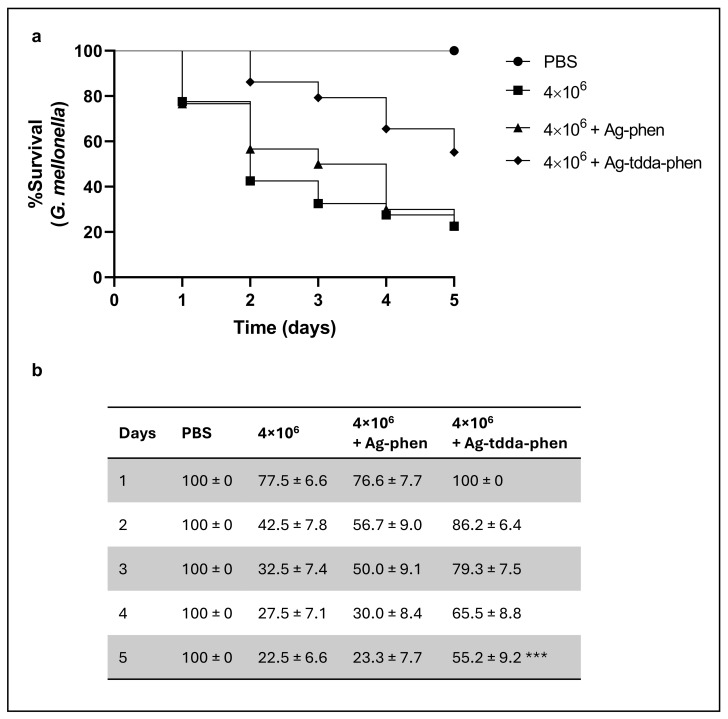
Effect of the Ag-phen and Ag-tdda-phen complexes on *G. mellonella* larvae infected with *F. pedrosoi*. Each system, consisting of a total of 10 larvae, was injected with 10 µL of a suspension containing *F. pedrosoi* conidia (4 × 10^6^) and non-cytotoxic concentrations of either Ag-phen (10 µg) or Ag-tdda-phen (5 µg). Untreated larvae, as well as those injected with only phosphate-buffered saline (PBS), were used as control groups. Larval mortality was monitored daily for five days and was determined based on dark pigmentation and the absence of movement in response to mechanical stimulation. (**a**) Kaplan–Meier survival curves and (**b**) representation of standard error values based on three independent experiments. Ag-tdda-phen significantly increased larval survival compared with untreated larvae (control group).*** *p* < 0.006, Log-rank (Mantel–Cox) test.

The ADMETlab 2.0 predicted that Ag-tdda-phen exhibits a favorable tolerability profile particularly as a topical antifungal agent. Although *G. mellonella* lacks anatomical similarity to human skin, its innate immune system shares several functional characteristics with that of humans [40]. For this reason, this insect has become a widely used preliminary model for assessing the toxicity and efficacy of antifungal compounds before studies in mammals [40,41]. Moreover, a recent study investigated the efficacy of several classical antifungal agents against CBM fungi using *G. mellonella* as in vivo model [42]. In this context, the efficacy of the silver(I) complexes on the survival of *G. mellonella* larvae after infection with *F. pedrosoi* conidia was also investigated. The results showed that after 5 days, fungal cells (4 × 10^6^) in the presence of 10 µL of the highest non-cytotoxic concentration of Ag-tdda-phen (500 mg/L) significantly increased larval survival (~2.5-fold) compared to untreated larvae (control group). In fact, while the survival percentage of untreated larvae was 22%, the treatment resulted in a 55% survival rate for *G. mellonella* larvae (Figure 6), suggesting a protective effect. Our results surpassed those observed in another study where *C. haemulonii* were pretreated with subinhibitory concentrations of the same silver(I) complexes and subsequently inoculated into *G. mellonella* larvae. After 48 h, Ag-phen and Ag-tdda-phen only increased larvae survival by 25 and 10%, respectively [16]. Similarly, a previous study from our group demonstrated a protective effect of [Ag(phendione)_2_]ClO_4_, which reduced the mortality of *G. mellonella* larvae infected with *P. verrucosa* [19]. For Ag-phen, even at the highest concentration tested (1000 mg/L), which did not affect larval viability, the compound failed to reduce mortality in the infected groups, displaying a survival profile similar to that of the control system (Figure 6).

Although further research is still required to fully elucidate the mechanisms through which metal-based complexes exert their antifungal activity, current evidence suggests that their mode of action is multimodal and strongly dependent on both the metal center and the coordinated ligands [43]. Although Ag-tdda-phen contains two phen ligands, studies by O’Shaughnessy et al. [15] demonstrated that antimicrobial activity is not necessarily determined by the number of coordinated phen ligands present in the complex. Therefore, we propose that the greater efficacy of Ag-tdda-phen compared to Ag-phen in reducing *F. pedrosoi*-induced larval mortality is attributable to its dual-ligand structure, comprising both phen and 3,6,9-tdda ligands. The tdda ligand contains three ether oxygen atoms and two carboxylate groups, which increase its polarity and aqueous solubility and potentially enhance its biodistribution within the larval hemolymph. The tdda ligand also stabilizes the metal center through efficient chelation, enabling a slower and more controlled release of Ag^+^ ions, which can prolong antifungal activity [15]. Moreover, the combination of tdda with phen creates a balanced heteroligand system, where tdda promotes solubility and biocompatibility, and phen facilitates penetration through lipid membranes and interaction with intracellular targets [15,44]. Such a physicochemical balance likely allows the complex to diffuse more effectively through larval tissues and reach the fungal cells. In addition, in vivo studies have suggested that tdda-based complexes may modulate the larval immune response, contributing to enhanced survival outcomes [15].

Based on our previous results with Ag-phen and Ag-tdda-phen [20], we propose that inhibition of biofilm and melanin formation by these compounds can increase *F. pedrosoi* susceptibility to interactions with *G. mellonella* larvae and macrophage-mediated killing, which is also influenced by ROS production, a key antimicrobial defense mechanism of phagocytic cells [37]. In addition, the ability of Ag(I) complexes to inhibit *F. pedrosoi* metallopeptidase activity [20] may impair the fungus’s capacity to acquire peptides and amino acids essential for nutrition, thereby restricting cell growth. Collectively, these potential modes of action may explain the reduced fungal burden observed following interactions with *G. mellonella* larvae and macrophages. Furthermore, the multifunctional nature of these compounds, particularly Ag-tdda-phen, which contains the tdda ligand whose chemical features enhance solubility, stability, and biological activity, may also contribute to the improved survival of larvae [15].

In this context, our current findings represent an important initial step toward identifying Ag-tdda-phen as a promising candidate for future testing in murine models. Metal complexes such as Ag-tdda-phen often exhibit mechanisms of action distinct from those of classical organic antifungal drugs while also offering advantages such as straightforward synthesis and low production costs.

## 3. Materials and Methods

### 3.1. Fungal Growth Conditions

*Fonsecaea pedrosoi* strain ATCC 46428 (also known as 5VPL), originally isolated from a Brazilian patient with CBM, was maintained on SDA medium at 4 °C for long-term preservation. For experimental use, the fungus was cultured in 100 mL of Czapek-Dox broth medium (BD-Difco, Silicon Valley, CA, USA), adjusted to pH 5.5, and incubated under constant agitation for 6 days at 26 °C. The cultures were filtered to obtain conidia and centrifuged at 2400× *g* for 10 min. The conidia were washed three times in 0.9% NaCl and the number of cells was determined after counting in a Neubauer chamber [20].

### 3.2. Compounds

In this study, the Ag-phen and Ag-tdda-phen complexes, which previously demonstrated anti-*F. pedrosoi* activity and the ability to modulate key biological processes in this fungus [20], were synthesized as described by McCann et al. [45] and Gandra et al. [14], respectively. Both compounds were dissolved in water and used for all experiments.

### 3.3. In Silico Analysis

The chemical structures of the compounds Ag-phen e Ag-tdda-phen were generated using Discovery Studio v20 (Accelrys, San Diego, CA, USA), and their predicted pharmacokinetic and toxicity properties were evaluated using ADMETlab 2.0 (https://admetlab3.scbdd.com/ (accessed on 25 March 2025)). Lipinski’s and Pfizer’s rules were applied to estimate drug-likeness and predict oral bioavailability [21,22]. The ADMET parameters were predicted for each complex, including absorption (HIA, F20%, F30%, Caco-2, and MDCK permeability), distribution (PPB, VD, BBB penetration, and Fu), metabolism (CYP1A2, CYP2C19, CYP2C9, CYP2D6, and CYP3A4 inhibition), excretion (CL and T½), and toxicity (hERG blockade, H-HT, DILI, Ames test, ROA, SS, and CP).

### 3.4. In Vitro and In Vivo Toxicity Assays

#### 3.4.1. Erythrocytes

Hemolytic activity was assessed using the microdilution method following incubation of sheep red blood cells (Novalab, Rio de Janeiro, RJ, Brazil) with Ag-phen and Ag-tdda-phen, as described by Evans et al. [46]. The complexes were added at concentrations ranging from 100 to 0.78 µM in a 96-well microplate containing PBS, pH 7.2. Subsequently, 2% sheep blood cells in PBS were added and the plate incubated at 37 °C for 24 h. After incubation, the systems were centrifuged at 500× *g* for 10 min a 4 °C, the supernatant was transferred to a new plate, and the absorbance values were assessed using a microplate reader (MULTISKAN SkyHigh; Thermo Scientific, Waltham, MA, USA) at 415 nm. Triton X-100 (1%), a nonionic surfactant, was used as the 100% hemolysis control, and a system containing only PBS was used as the 100% viability control. The CC_50_ values were calculated, and the SI was calculated using the following equation: SI = erythrocyte CC_50_/*F. pedrosoi* IC_50_.

#### 3.4.2. Macrophage Cells

The murine macrophage cell line RAW 264.7 obtained from the ATCC was used for this set of experiments. The cells were cultured in 25 cm^2^ flasks (TPP Techno Plastic Products AG, Trasadingen, Switzerland) containing “Roswell Park Memorial Institute” medium (RPMI, Sigma-Aldrich, St. Louis, MO, USA) supplemented with 10% fetal bovine serum (Gibco, Grand Island, NY, USA) at 37 °C in an atmosphere of 5% CO_2_ and the cultures maintained in the logarithmic growth phase [19]. For the assays, 1 × 10^5^ macrophages were added to a 96-well flat-bottomed microplate containing RPMI medium and incubated in the absence (control) or presence of Ag-phen and Ag-tdda-phen at concentrations ranging from 160 to 1.25 μM. After 20 h of incubation, macrophage viability was assessed using a colorimetric MTT assay (Sigma-Aldrich, St. Louis, MO, USA). For that, 5 mg/mL of MTT in PBS, pH 7.2, was added to each well and the plate incubated in the dark for 3 h at 37 °C in 5% CO_2_. The systems were centrifuged at 1500× *g* for 7 min and the crystals were solubilized in sodium dimethyl sulfoxide (Sigma-Aldrich, St. Louis, MO, USA) before reading at 490 nm using a spectrofluorimeter FlexStation 3 (Molecular Devices, San Jose, CA, USA) [47]. The SI values were calculated as previously described for erythrocyte cells.

#### 3.4.3. Insect Larvae

For each experimental system, a total of 10 larvae of *T. molitor* or *G. mellonella* exhibiting a clear and uniform coloration and weighing approximately 0.15 g and 0.25 g, respectively, were selected. To evaluate toxicity, an inoculum containing 120, 250, 500, or 1000 mg/L of each complex (Ag-phen and Ag-tdda-phen) was injected into the last left proleg of each larva using an insulin syringe (BD Ultra-Fine, Franklin Lakes, NJ, USA). Then, the systems were incubated in the dark at 37 °C inside Petri dishes. Systems containing larvae injected only with sterile PBS were used as controls. The mortality rate was monitored daily for 5 days (120 h) and determined considering the dark pigmentation and lack of movement of the larvae in response to mechanical stimulus [19,48].

### 3.5. Effect of Compounds on the In Vitro Interaction of F. pedrosoi and Macrophages

#### 3.5.1. Adhesion and Killing Assays

For cell interaction assays, macrophages (1 × 10^5^ cells/mL) obtained as detailed above were incubated with *F. pedrosoi* conidia (5 × 10^5^ cells/mL) at a ratio of 1:10 (macrophage/conidia) in 24-well microplates (Costar, Corning Incorporated, Corning, NY, USA). After 1 h of interaction, non-adhered fungi were removed by washing in PBS and macrophages with adhered and/or internalized fungi were subjected to treatment with non-cytotoxic concentrations of either Ag-phen or Ag-tdda-phen. A control system containing only macrophages and fungi was also prepared. After 20 h of incubation at 37 °C in a 5% CO_2_ atmosphere, the systems were washed twice with PBS and the adhesion index determined after fixation and staining with Giemsa (Sigma-Aldrich, St. Louis, MO, USA), as described by Granato et al. [19]. In parallel, the killing capacity of macrophages was evaluated after lysing with sterile ice-cold water and plating on SDA medium. After 6 days of incubation at 26 °C, the number of viable conidia was quantified using the spread plate technique to determine the number of CFUs [19].

#### 3.5.2. Macrophage ROS Production

The effect of the compounds on macrophage microbicidal activity was performed in flat-bottom black polystyrene 96-well microplates with micro-clear bottoms (Greneir Bio One GmbH, Frickenhausen, Germany). Briefly, uninfected and *F. pedrosoi*-infected phagocytes that were not treated or treated with sub-inhibitory concentrations of Ag-phen and Ag-tdda-phen were incubated with 10 µM of the probe dichlorofluorescein diacetate (Sigma-Aldrich, St. Louis, MO, USA). The fluorescence was monitored using a FlexStation 3 spectrofluorimeter (Molecular Devices, San Jose, CA, USA) at an excitation wavelength of 485 nm and emission wavelength of 530 nm for 2 h after incubation. Systems treated with 1 mM H_2_O_2_ and the antioxidant NAC (15 mM) (Sigma-Aldrich, St. Louis, MO, USA) were used as the positive control and as an ROS production inhibitor, respectively [49,50].

### 3.6. Effect of Compounds on G. mellonella Infected with F. pedrosoi Conidia

For the efficacy evaluation of compounds, 10 µL of a suspension containing 4 × 10^6^ conidia either without (control) or with non-cytotoxic concentrations of Ag-phen or Ag-tdda-phen was injected into each group of 10 larvae. Larval mortality was monitored daily until the fifth day and was determined based on the absence of movement in response to mechanical stimuli and by dark pigmentation [19].

### 3.7. Statistical Analysis

The values presented as results represent the average of independent experiments in triplicate. The calculations and graphs of this study were performed using the “GraphPad Prism” program version 8.0.1. For statistical analysis were used the One-way ANOVA test, which stipulated significant values when *p* was equal to or less than 0.05. The survival curves were obtained according to Kaplan–Meier and compared using the Log-Rank (Mantel–Cox) test of “Graph Pad Prism” version 8.0.1.

## 4. Conclusions

Overall, these experiments showed that the silver(I) complexes Ag-phen and Ag-tdda-phen had favorable tolerability profiles in the in silico predictions, in vitro using erythrocytes and macrophage cells, as well as in vivo with *G. mellonella* and *T. molitor*. Moreover, both complexes were able to reduce the viability of *F. pedrosoi* conidia after macrophage interaction. Both complexes, especially Ag-tdda-phen, increased macrophage ROS levels, indicating that they can induce oxidative stress, which contributes to the phagocyte killing response. In addition, only Ag-tdda-phen was able to reduce *G. mellonella* larvae mortality following infection with *F. pedrosoi*. Combined with our previous results [20], we believe that our current findings corroborate the hypothesis that silver(I)-phen complexes represent a therapeutic option for CBM caused by *F. pedrosoi*. There are ongoing experiments with other CBM fungi and with the purpose of clarifying the mechanisms of action of these silver(I) complexes.

## Figures and Tables

**Table 1:** Predicted ADMET profiles of silver(I)-1,10-phenanthroline complexes and the reference antifungal itraconazole.

Pharmacokinetic Property	Compounds		
	Ag-tdda-phen	Ag-phen	Itraconazole
Lipinski’s violation	Yes (≥2)	No	Yes (≥2)
Pfizer’s violation	No	No	No
HIA (%)	Low (1.0)	Low (0.98)	High (0.002)
F20%	Low (1.0)	Low (1.0)	High (0.002)
F30%	Low (1.0)	Low (1.0)	High (0.008)
CaCo-2 (Log cm/s)	Low (−5.976)	Low (−6.077)	High (−5.091)
MDCK (cm/s)	High (0.00027)	High (0.00019)	ND
PPB (%)	Low (62.17)	Low (58.82)	High (98.01)
VD (L/kg)	High (0.404)	High (1.413)	High (2.043)
BBB penetration (cm/s)	No (0.899)	Moderate (0.646)	Yes (0.011)
Fu (%)	(No) 8.144	(No) 12.419	Yes (0.315)
CYP1A2 inhibition	No (0.0)	No (0.0)	Yes (0.097)
CYP2C19 inhibition	No (0.0)	No (0.0)	Yes (0.697)
CYP2C9 inhibition	No (0.0)	No (0.001)	Yes (0.965)
CYP2D6 inhibition	No (0.0)	No (0.0)	Yes (0.112)
CYP3A4 inhibition	No (0.0)	No (0.0)	Yes (0.975)
CL *plasma* (mL/min/kg)	Low (0.612)	Low (1.732)	Moderate (8.329)
T1/2	Low (0.423)	Low (0.563)	Low (0.324)
hERG blockade	No (0.14)	No (0.008)	Yes (0.865)
H-HT	Yes (1.0)	Moderate (0.32)	Yes (0.989)
DILI	Yes (0.99)	Yes (0.99)	Yes (0.995)
AMES mutagenicity	Moderate (0.604)	High (0.862)	High (0.924)
ROA toxicity	Low (0.183)	High (0.984)	Moderate (0.694)
SS	Yes (0.981)	Yes (0.991)	Yes (0.843)
CP	Yes (0.99)	Yes (0.969)	Yes (0.818)

Caco-2: human colon adenocarcinoma cells; MDCK: Maden Darby Canine Kidney cells; PPB: plasma protein binding; VD: volume distribution; BBB: blood–brain barrier; Fu: unbound fraction; CYP: cytochrome P450 isoenzymes; CL: clearance; T½: half-life; hERG: human ether-à-go-go-related gene; H-HT: human hepatotoxicity; DILI: drug-induced liver injury; ROA: rat oral acute; SS: skin sensitization; CP: carcinogenic potency; ND: not determined.

**Table 2:** Selectivity index of silver(I)-1,10-phenanthroline complexes.

Compound	CC_50_ (µM) Erythrocytes	CC_50_ (µM) RAW	IC_50_ (µM) *F. pedrosoi*	SI Erythrocytes	SI RAW
Ag-phen	17.01	>10.0	0.62	27.4	>16.1
Ag-tdda-phen	10.03	>10.0	0.31	32.3	>32.2

The selectivity index (SI) was calculated as the ratio between the concentration of the compound required to inhibit erythrocyte or macrophage viability by 50% (CC_50_) and the concentration required to inhibit *F. pedrosoi* growth by 50% (IC_50_).

## Data Availability

The original contributions presented in this study are included in the article. Further inquiries can be directed to the corresponding author.

## Abbreviations

The following abbreviations are used in this manuscript:

Ag-phen	[Ag(1,10-phenanthroline)_2_]ClO_4_
Ag-tdda-phen	[Ag_2_(3,6,9-trioxaundecanedioate)(1,10-phenanthroline)_4_]·EtOH
BBB	Blood–brain barrier
CaCo-2	Human colon adenocarcinoma
CBM	Chromoblastomycosis
CL	Clearance
CP	Carcinogenic potency
CYP	Cytochrome P450
DILI	Drug-induced liver injury
Fu	Fraction unbound
hERG	Human ether-à-go-go-related gene
HIA	Human intestinal absorption
H-HT	Human hepatotoxicity
MIC	Minimum inhibitory concentration
MDCK	Maden Darby canine kidney
MFC	Minimum fungicidal concentration
NAC	*N*-acetylcysteine
PBS	Phosphate-buffered saline
Phen	1,10-phenantholine
Phendione	1,10-phenantholine-5,6-dione
PPB	Plasma protein binding
RPMI	Roswell Park Memorial Institute
ROA	Rat oral acute
ROS	Reactive oxygen species
SDA	Sabouraud Dextrose Agar
SI	Selectivity index
SS	Skin sensitization
T½	Half-life
TPSA	Topological polar surface area
VD	Volume of distribution
WHO	World Health Organization
